# Disruption of a rice gene for α-glucan water dikinase, *OsGWD1*, leads to hyperaccumulation of starch in leaves but exhibits limited effects on growth

**DOI:** 10.3389/fpls.2013.00147

**Published:** 2013-05-27

**Authors:** Tatsuro Hirose, Naohiro Aoki, Yusuke Harada, Masaki Okamura, Yoichi Hashida, Ryu Ohsugi, Miyao Akio, Hirohiko Hirochika, Tomio Terao

**Affiliations:** ^1^NARO Agricultural Research CenterNiigata, Japan; ^2^Graduate School of Agricultural and Life Sciences, The University of TokyoTokyo, Japan; ^3^National Institute for Agrobiological SciencesIbaraki, Japan

**Keywords:** assimilate partitioning, gene disruption mutant, α-glucan water dikinase, leaf starch, rice

## Abstract

To identify potential regulators of photoassimilate partitioning, we screened for rice mutant plants that accumulate high levels of starch in the leaf blades, and a mutant line leaf starch excess 1 (LSE1) was obtained and characterized. The starch content in the leaf blades of LSE1 was more than 10-fold higher than that in wild-type plants throughout the day, while the sucrose content was unaffected. The gene responsible for the LSE1 phenotype was identified by gene mapping to be a gene encoding α-glucan water dikinase, *OsGWD1* (Os06g0498400), and a 3.4-kb deletion of the gene was found in the mutant plant. Despite the hyperaccumulation of starch in their leaf blades, LSE1 plants exhibited no significant change in vegetative growth, presenting a clear contrast to the reported mutants of *Arabidopsis thaliana* and *Lotus japonicus* in which disruption of the genes for α-glucan water dikinase leads to marked inhibition of vegetative growth. In reproductive growth, however, LSE1 exhibited fewer panicles per plant, lower percentage of ripened grains and smaller grains; consequently, the grain yield was lower in LSE1 plants than in wild-type plants by 20~40%. Collectively, although α-glucan water dikinase was suggested to have universal importance in leaf starch degradation in higher plants, the physiological priority of leaf starch in photoassimilate allocation may vary among plant species.

## INTRODUCTION

Assimilate partitioning has long been recognized as a target for crop improvement because it can limit the yield potential of the crop plants. Despite the great importance of rice as a major crop, most of the mechanisms of photoassimilate allocation in rice are yet to be elucidated. Utilizing mutants that exhibit phenotypes relevant to a particular biological phenomenon can be a powerful approach to uncovering potential regulators of that phenomenon. In the source leaves of higher plants, both starch and sucrose are the major primary products of photosynthesis; therefore any anomaly in the accumulation of the two compounds may arise from mutations in the genes involved in assimilate allocation. In particular, the accumulation of an extraordinarily high level of leaf starch is easily recognizable by iodine staining, and thus has been repeatedly reported as the “starch excess” phenotype. Starch excess in the leaves is often observed when any inhibition occurs (1) in the export of photoassimilate from source leaves or (2) in starch metabolism in the leaves. The former case includes disruption or suppression of the phloem-loading sucrose transporter (Bürkle et al., 1998; Gottwald et al., 2000) and blockade of the route of assimilate transport (Russin et al., 1996). Cold-girdling of the stem is also known to induce the starch excess phenotype through the impairment of photoassimilate transport (e.g., Krapp et al., 1993). As an instance of the latter case, the *Arabidopsis thaliana* Starch EXcess 1 (SEX1) mutant (Caspar et al., 1991) is the most well-known because of its significant contribution to the current understanding of leaf starch metabolism, i.e., α-glucan water dikinase (GWD, EC 2.7.9.4)-mediated degradation. Disruption of the plastid-localized maltose translocator (Niittylä et al., 2004) is also known to result in starch excess in the leaf, as well as some other mutations of starch degradation-related enzymes (Critchley et al., 2001). As is clear from the above described examples, the study of mutant plants exhibiting the starch excess phenotype in their leaves is a good approach to acquiring a better understanding of the mechanisms and regulation of photoassimilate allocation.

Thus far, no rice mutant that accumulates a high level of starch in the leaf blades has been reported. However, in our preliminary experiments, we found that pronounced starch accumulation occurred in detached leaf blades when sucrose was fed through their cut ends. This finding suggested that a rice mutant exhibiting the starch excess phenotype could exist and that it might be caused by a disorder in the mechanism controlling photoassimilate partitioning. We therefore decided to screen for mutant lines accumulating high levels of starch in their leaf blades. Here we report the isolation and characterization of the first leaf starch excess mutant of rice.

## MATERIALS AND METHODS

### SCREENING OF THE LEAF STARCH EXCESS MUTANTS

In this study, we used a collection of rice mutant lines induced by the insertion of the endogenous retrotransposon Tos17 (Miyao et al., 2003). To screen for mutants displaying the leaf starch excess (LSE) phenotype, 20 seeds of the M2 or M3 generation of Tos17 mutant lines were grown to the fourth leaf stage in the greenhouse. The terrestrial parts were sampled in the morning and leaf starch was visualized by iodine staining.

### GENE MAPPING

Mapping populations were obtained from crosses between mutants with the *japonica *background and an *indica*/*japonica* crossbred rice cultivar “Takanari.” In the F2 generation, mutant plants were selected by iodine staining of the terrestrial parts. DNA was extracted according to the method reported by Wang et al. (1993) from the leaf segments of plants showing the mutant phenotype. Gene mapping was carried out using micro-satellite SSR markers (McCouch et al., 2002).

### ANALYSIS OF THE GENE STRUCTURE

Genomic DNA was extracted from green leaves by using the DNeasy Plant Kit (Qiagen, Valencia, CA, USA) and was used as the template for polymerase chain reaction (PCR) with PrimeStar GXL DNA Polymerase (Takara Bio Inc., Shiga, Japan) following the manufacturer’s instructions. The nucleotide sequences of the PCR primers are listed in **Table 1**. For the analysis of the transcript structures, total RNA was extracted from the leaf blades and reverse-transcribed as below, and the resultant cDNA was used as the template for PCR with ExTaq DNA polymerase (Takara Bio Inc.).

**Table 1:** List of the PCR primers used in this study.

Gene	Primer	Nucleotide sequence	Purpose*
*OsGWD1*	PL2	ttgccttctgttcgccttaaaa	C
	L4	ctctcccaaggtactgggt	I, S
	L17	tgaagccacgtgagataagc	S
	L18	gcttaaagggatggaatcaagc	S
	L19	gcagaagctggccaggcagt	R
	R2	gttatacatgtcccacggc	I
	R14	aacattgcctgattgacttggcta	C, S
	R23	ctgccaacttccaagctg	S
	R24	acgttgcgagacttggcccc	R
*RUBIQ1*	L1	ggagctgctgctgttcttgg	R
	R1	cacaatgaaacgggacacga	R

* *C, gene construct for functional complementation; I, gene construct for RNAi suppression; R, real-time RT-PCR; S, analysis of the transcript structure*.

### GENERATION OF TRANSGENIC RICE PLANTS

For complementation analysis a 13.2-kb genomic DNA fragment containing the entire open reading frame (ORF) along with 2.2-kb sequence upstream of the putative translation start point of *OsGWD1* (Os06g0498400) was amplified by PCR using the primer pair, GWD1-PL2/R14 (**Table 1**), genomic DNA of the rice cultivar Nipponbare (as the template), and PrimeStar GXL DNA polymerase (Takara Bio Inc.). The DNA fragment was introduced into a transformation vector, pZH2B (Kuroda et al., 2010). For gene suppression analysis, a 0.4-kb fragment of *OsGWD1* was amplified using ExTaq DNA polymerase (Takara Bio Inc.), with the GWD1-L4/R2 primer pair (**Table 1**) and cDNA from green seedlings, and introduced into the RNAi transformation vector, pANDA (Miki and Shimamoto, 2004). Transgenic rice plants were generated using the *Agrobacterium tumefaciens*-mediated method described by Hiei et al. (1994), and grown in a temperature-controlled glasshouse (30/23°C, day/night).

### QUANTITATIVE RT-PCR ANALYSIS

For the analyses of mRNA abundance, rice plants were grown in plastic pots filled with nursery soil for rice seedlings. For tissue-specific expression analysis, rice seedlings were grown in a greenhouse under natural light conditions. For expression analyses of diurnal changes, rice plants were grown in a growth chamber with a 14-h light (27°C)/10-h dark (23°C) photoperiod. The light intensity in the light period was 350 μmol m^-^^2^ s^-^^1^ and the light was emitted by fluorescent light tubes. Once the tissue samples were obtained from the plants, they were immediately frozen in liquid nitrogen and stored at -80°C until use. Total RNA was extracted from various tissue samples using the extraction buffer described by Chang et al. (1993), although polyvinylpyrrolidone and spermidine were excluded. The extracted RNA was then purified using an RNeasy Mini Kit (Qiagen) followed by a TURBO DNA-free Kit (Ambion, Austin, TX, USA) to remove DNA. The purified RNA was reverse-transcribed and then an aliquot of the first-strand cDNA mixtures corresponding to 5 ng of total RNA was used as the template for real-time quantitative reverse transcription (RT)-PCR analysis using the SuperScriptIII Platinum Two-Step qRT-PCR Kit with SYBR Green (Life Technologies, Austin, TX, USA). The reaction was carried out using an ABI7300 system (Applied Biosystems, Foster City, CA, USA) with the gene-specific primers listed in **Table 1**. In preliminary experiments, aliquots of the PCR reactions were electrophoretically separated on agarose gels to verify the specificity of the primers. The specificity of each PCR amplification was also checked using a heat dissociation protocol with temperatures changing from 60 to 95°C following the final cycle of the PCR. The results obtained for each cDNA were standardized to the expression of the rice polyubiquitin gene (*RUBIQ1*), which is a constitutively expressed gene in rice (Wang et al., 2000).

### DETERMINATION OF STARCH AND SOLUBLE SUGAR CONTENTS

Frozen leaf samples were ground using a mortar and pestle under cryogenic conditions. A portion of the ground tissue (~25 mg per sample) was immediately weighed and then extracted twice with 80% ethanol at 80°C. After centrifugation at 8500 *g* for 5 min, the supernatant was dried *in vacuo*, dissolved in distilled water, and used to assay sucrose, glucose, and fructose by the enzymatic method using F-kit #716260 (J. K. International, Tokyo, Japan), which contains an invertase, a hexokinase, a glucose 6-phosphate dehydrogenase (G6PDH), and a phosphoglucomutase. The residual pellet obtained after centrifugation was dried in air, resuspended in distilled water, gelatinized by boiling for 4 h, and used to assay starch by the enzymatic method using F-kit #207748 (J. K. International), which contains an amyloglucosidase, a hexokinase, and a G6PDH. The assays were conducted according to the manufacturer’s instructions, and the increase in the absorbance at 340 nm in each assay, resulting from the enzymatic conversions of starch, sucrose, glucose, or fructose into 6-phosphogluconate, were measured with a 96-well plate reader (Viento XS; Dainippon Sumitomo Pharma, Osaka, Japan).

### DETERMINATION OF THE PHOSPHORYLATION STATUS OF STARCH

Fully elongated leaf blades of 3-week-old seedlings, grown under natural light conditions, were sampled 2 h after sunset, and frozen immediately. Approximately 200 mg of frozen leaves were used in order to extract the starch, using the method as described above with slight modifications. The pellet after centrifugation was washed with distilled water three times to remove soluble metabolites completely, and resuspended in 0.5 mL distilled water. The resuspended pellet was gelatinized by boiling for 4 h; then, it was digested thoroughly to hexose units by adding 0.1 mL amyloglucosidase (TOYOBO, Tokyo, Japan) at a concentration of 50 U mL^-^^1^ in 50 mM sodium acetate buffer (pH 4.6) and incubating the solution at 60°C for 18 h with gentle shaking. Using the resultant digested starch samples, we first measured the amount of glucose, [Glc]_starch_, by the same enzymatic method as described above. Next, as for the same digested starch samples, we also measured the amount of glucose 6-phosphate, [G6P]_starch_, by another enzymatic method using highly purified G6PDH (Roche Diagnostics, Mannheim, Germany) as the sole enzyme, to allow the conversion of only G6P to 6-phosphogluconate. More than 30 μg of starch (equivalent to 185 nmol of the hexose unit) was used to assay G6P in a 200-μL reaction, in order to obtain significant increases in the absorbance at 340 nm. No significant changes in the absorbance were detected when 200 nmol of authentic glucose instead of digested starch samples were added into the 200-μL assay mixture, indicating that the hexokinase activity contamination from the G6PDH enzyme solution was negligible.

Since the enzymatic assay of [Glc]_starch_ provides the total concentration of Glc plus G6P, the phosphorylation status of starch was calculated according to the following equation:

$${\left[G6P\right]}_{starch}/{\left[Glc\right]}_{starch}\times 100\,=\text{percentage of phosphorylated glucose residue in starch (\%).}$$

### MEASUREMENTS OF PHOTOSYNTHESIS RATE AND ^13^C-PHOTOASSIMILATE PARTITIONING

Rice plants were grown in plastic pots in glasshouses until the 6th to 7th leaf stage and then used for the experiments. Net CO_2_-assimilation rates in the leaf blades were measured using a portable photosynthesis system (LI-6400, LI-COR, Lincoln, NE, USA). Analysis of ^13^C-photoassimilate partitioning in rice plants was conducted according to the method described by Sasaki et al. (2005) with modifications. In brief, ^13^CO_2_ was fed to rice seedlings in sealed, transparent plastic box, for 2 h (from 11:00 to 13:00) under the light intensity of around 750 μmol m^-^^2^ s^-^^1^, in a controlled glasshouse (27/23°C, 14-h day/10-h night, the day period from 5:00 to 19:00). ^13^CO_2_ gas was liberated from Ba^13^CO_3_ powder (≥98 atom%, Cambridge Isotope Laboratories, Andover, MA, USA) mixed with 7.3 M H_3_PO_4_ inside the box. After the ^13^C-feeding was ceased by removing the plastic box, the aboveground parts of the seedlings were sampled at designated times; immediately after the cessation of ^13^C-feeding (13:00), in the evening of the same day (19:00), and in the morning of the next day (7:00). The aboveground samples were divided into leaf blades and sheaths, and then stored at -80°C until use. For measuring ^13^C contents, the frozen tissue samples were freeze-dried, followed by measuring dry weight. The dried tissue materials were ground to a fine powder, and a portion (ca. 200 μg) of the tissue powder was used for the determination of ^13^C content. The total carbon and ^13^C contents were determined using an elemental analyzer (NC2500, Thermoquest, San Jose, CA, USA) and a mass spectrometer (Delta Plus System, Thermoquest). The ^13^C content in each tissue sample was calculated according to the equation of Sasaki et al. (2005). Partitioning of ^13^C-photoassimilates to the leaf blades was calculated from the total amounts of ^13^C in leaf blades, [^13^C]_LB_, and sheaths, [^13^C]_LS_, according to the following equation:

[C13]LB/{[C13]LB+[C13]LS}×100=C13−partitioning to leaf blades (%).

### FIELD TRIALS AND DETERMINATION OF YIELD COMPONENTS AND GRAIN YIELD

Field trials were carried out in paddy fields at the Institute for Sustainable Agro-ecosystem Services (ISAS), Tokyo, Japan (35°44^′^N, 139°32^′^E). Seeds were sown in a greenhouse in late April, and transplanted into the paddy fields in late May. In any given year, the planting density was 22.2 hills per square meter (hill spacing of 30 cm × 15 cm) with one seedling per hill, and compound fertilizer for paddy fields (N:P_2_O_5_:K_2_O = 12:16:18%) was applied at the rate of 50 g m^-^^2^ as a basal dressing. Approximately 45 days after the heading, plants were harvested in late September, and their panicles were used for the analysis of yield components. The panicle number per hill (A) and spikelet number per panicle (B) were counted by hands. Subsequently, ripened grains were selected as unhulled grains that settle down in a salt solution with a density of 1.06 g mL^-^^1^. The percentage of ripened grains (C) was calculated from the total spikelet number per hill and the ripened grain number. Unhulled grain yield was calculated from the yield components measured and the planting density according to the following equation.

$$A\,\times B\times C/100\times [\text{grain weight }\left({g\,1000-grain\,}^{-1}\right)]/1000\times 22.2({hill\,m}^{-2})=\text{grain yield }\left({g\,m}^{-2}\right).$$

## RESULTS

### SCREENING OF THE LEAF STARCH EXCESS MUTANTS AND THE LSE1 PHENOTYPE

**FIGURE 1:**
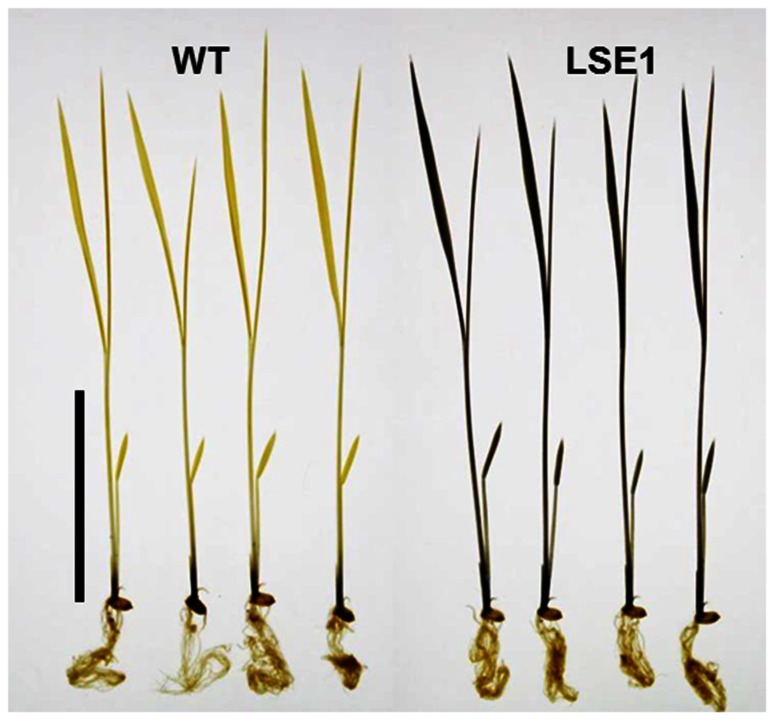
**Iodine staining of LSE1 mutant seedlings**. Rice seedlings were grown for 14 days and sampled just after the onset of light period. Starch was visualized by iodine staining after the chlorophyll was removed by ethanol. Bar=5cm

**FIGURE 2:**
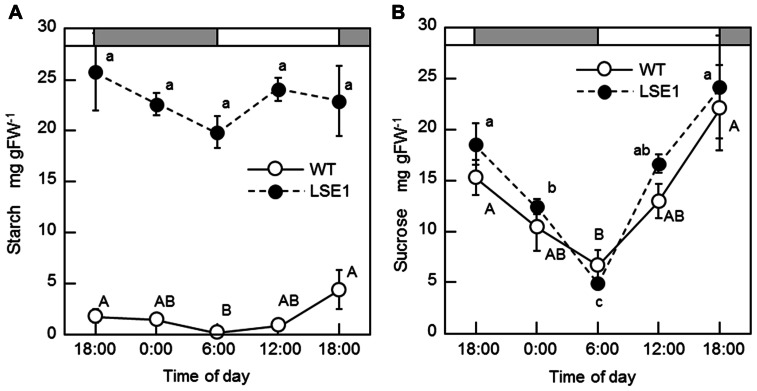
**Diurnal changes in starch (A) and sucrose (B) levels**. Rice seedlings were grown in a growth chamber with a 12-h light (6:00–18:00)/12-h dark photoperiod. Temperature was maintained at 25°C during the light period and at 20°C during the dark period. White and gray bars represent light and dark periods, respectively. Fully expanded leaf blades of 3-week-old seedlings were harvested at designated times of the day and used for the measurements. Data represent the mean ± SE (*n* = 5) and the different letters indicate significant differences within each of the two lines, capital letters for WT and small letters for LSE1 (*p* < 0.05, Tukey’s test).

Of 6377 Tos17 mutant lines, 5 were selected by iodine staining to detect distinct accumulation of leaf starch (stain-positive plants). One of the five lines designated as ND0717, exhibited dark staining for starch in the entire terrestrial portion while the wild-type plants did not show any discernible staining (**Figure 1**). Because the seeds originally used for the screening were M2 seeds, i.e., those borne on the M1 generation plants, they segregated into stain-positive and stain-negative plants. The progeny of these M2 plants were further tested for leaf starch accumulation, and in the M3 generation, the progeny of some stain-negative M2 plants segregated into stain-positive and stain-negative ones at a ratio of 1:3, suggesting that the LSE phenotype was due to the recessive mutation of a single gene. To establish homozygously mutated lines, the progeny of stain-positive M2 plants were examined to check whether all the individuals showed the stain-positive phenotype. Based on the results of this analysis, of the 15 original M2 plants, 2 were identified as having the homozygous mutant genotype, 2 as wild-type, and the remaining 11 as having the heterozygous genotype. After this, we used the progeny of the two putative homozygous mutant plants as the pure line for the mutation and designated this mutation LSE1, while the progeny of the two putative wild-type genotype plants were used as controls in the various assays in this study and were designated “wild-type.” The starch content in the mature leaf blades of LSE1 plants was 5- to 10-fold higher than that in the wild-type plants throughout the day (**Figure 2A**). Contrastingly, the sucrose content in the leaf blades was not different between the two (**Figure 2B**).

### GENE MAPPING OF THE LSE1 MUTATION

**FIGURE 3:**
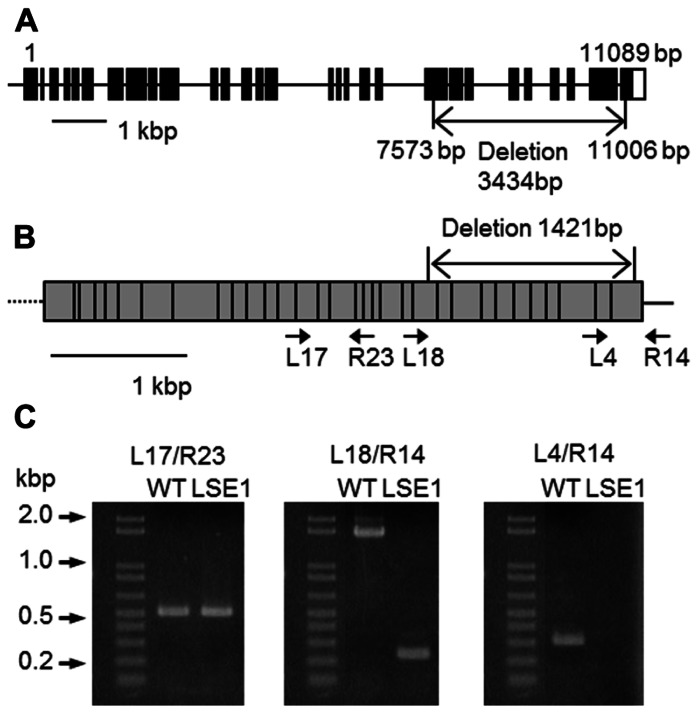
**Structure of *OsGWD1* and its transcript**. **(A)** The gene structure of *OsGWD1* is illustrated; black boxes indicate exons and the position of the gene deletion in LSE1 mutant is shown by an arrow. **(B)** The coding region of the *OsGWD1* transcript is indicated by a gray rectangle, in which the junctions of the exons are shown by vertical bars. The positions of the PCR primers used for the analysis of the transcript structure are indicated by the arrow heads (see below). Note that the primers L17, L18, L4, and R23 span the exon/intron junction on the cDNA. **(C)** Agarose gel electrophoresis images of the PCR products are presented to demonstrate that an aberrant transcript is accumulated in the leaves of LSE1 plants. The results of PCR with the different primer pairs are shown.

Southern blot analysis revealed that the LSE phenotype of LSE1 was not tagged by the Tos17 retrotransposon; it was therefore believed to be caused by some other mutation (data not shown). In order to identify the responsible gene for the mutation, gene mapping was carried out. The responsible gene was mapped to a 1.16-Mb region on chromosome 6 flanked by two genetic markers RM20061 and RM20104 (McCouch et al., 2002). Although as many as 77 genes are annotated within this region, it was significant that a gene for a putative GWD, Os06g0498400, was located in this region because disruption of the corresponding orthologs of this gene results in the LSE phenotype in both *Arabidopsis* (SEX1 mutant; Caspar et al., 1991) and *Lotus japonicus* (Vriet et al., 2010). Therefore, we decided to further explore this gene as a major candidate gene causing the LSE1 phenotype, and the gene Os06g0498400 was designated as *OsGWD1*. As the first step, we examined whether the structure of the *OsGWD1* gene was different between the LSE1 mutant and the original cultivar Nipponbare, by genomic PCR analysis using several primer combinations designed based on the published DNA sequence of Os06g0498400. *OsGWD1* has been predicted to be a relatively complex gene structure comprising as many as 32 exons, and it was found that the *OsGWD1* gene in the LSE1 mutant had a long deletion of 3434 bp corresponding to the nucleotide position from 7415 to 10758 of the reference sequence in the database, NC_008399. As a result, *OsGWD1* in the LSE1 mutant lacks the exons from the 22nd to the 32nd (**Figure 3A**).

### EXPRESSION ANALYSIS OF *OsGWD1* IN LSE1 AND THE WILD-TYPE PLANTS

The transcript of *OsGWD1* in the leaf blades was analyzed in both LSE1 and the original cultivar Nipponbare by RT-PCR. According to the gene structure and the sequences of the cDNA clones in the database, the transcript of *OsGWD1* was predicted to be ~4.4 kb in wild-type plants and to contain a 1421-bp deletion in the case of LSE1, and the primer pairs were designed to check the structure of the transcript (**Figure 3B**). With a primer pair that spanned the LSE1 deletion (L18/R14; **Figure 3C**), a shorter PCR product was amplified with the expected size in LSE1, and no PCR product was detected with a left primer that was located within the deletion (L4/R14; **Figure 3C**).

**FIGURE 4:**
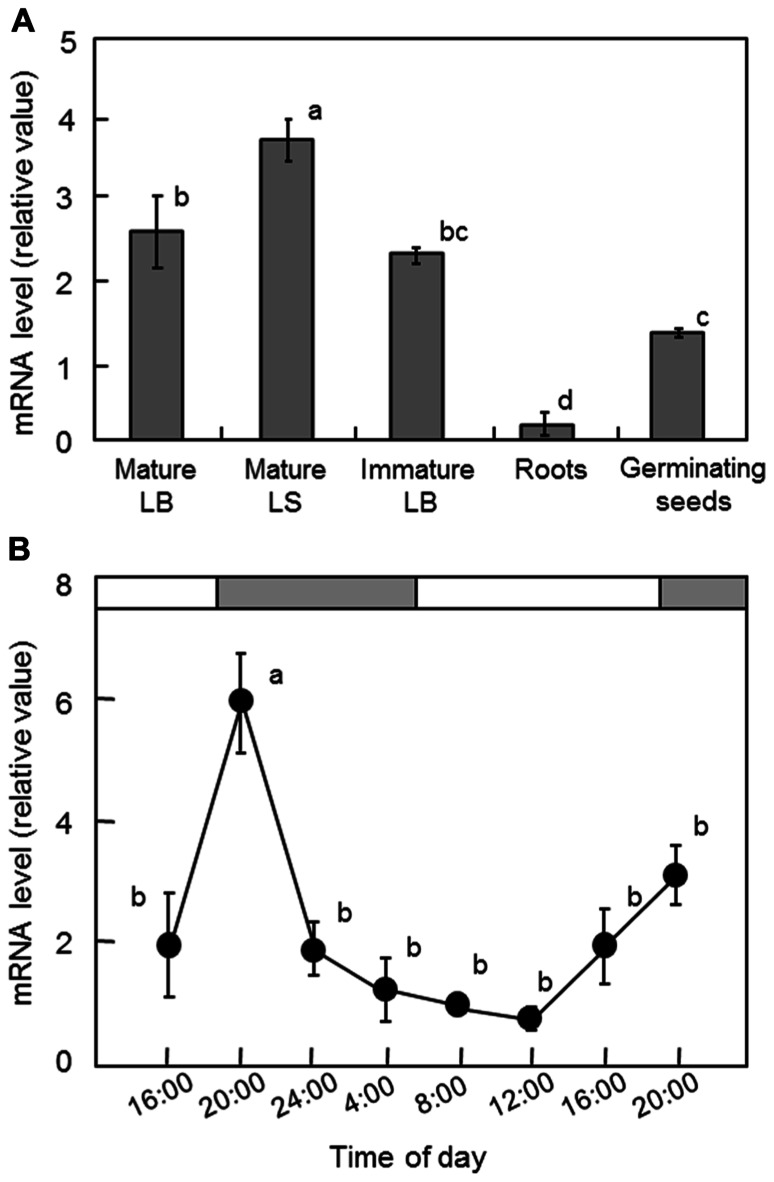
**Transcript levels of *OsGWD1* in rice seedlings**. Tissue-specific accumulation pattern **(A)** and diurnal changes in leaf blades **(B)**, with respect to the mRNA levels of *OsGWD1* were examined by quantitative RT-PCR analysis. Values are standardized to the expression level of a rice polyubiquitin gene (% *RUBIQ1*). Data represent the mean ± SE (*n* = 4) and the different letters indicate significant differences (*p* < 0.05, Tukey’s test). LB, leaf blade; LS, leaf sheath.

To determine the expression pattern of *OsGWD1* in normal rice plants, real-time RT-PCR analysis was conducted using various tissues from 2-week-old plants and 4-day-old germinating seeds of Nipponbare. The mRNA levels in both leaf blades and sheaths were relatively high, while those in the roots were much lower (**Figure 4A**). The diurnal change in the mRNA level of *OsGWD1* was also examined using the fully expanded leaf blades of 2-week-old plants, grown with a 14-h light period (**Figure 4B**). The mRNA level of *OsGWD1* was highest in the early night (20:00) and then decreased until the next noon (12:00).

### FUNCTIONAL COMPLEMENTATION OF *OsGWD1* IN THE LSE1 MUTANT

**FIGURE 5:**
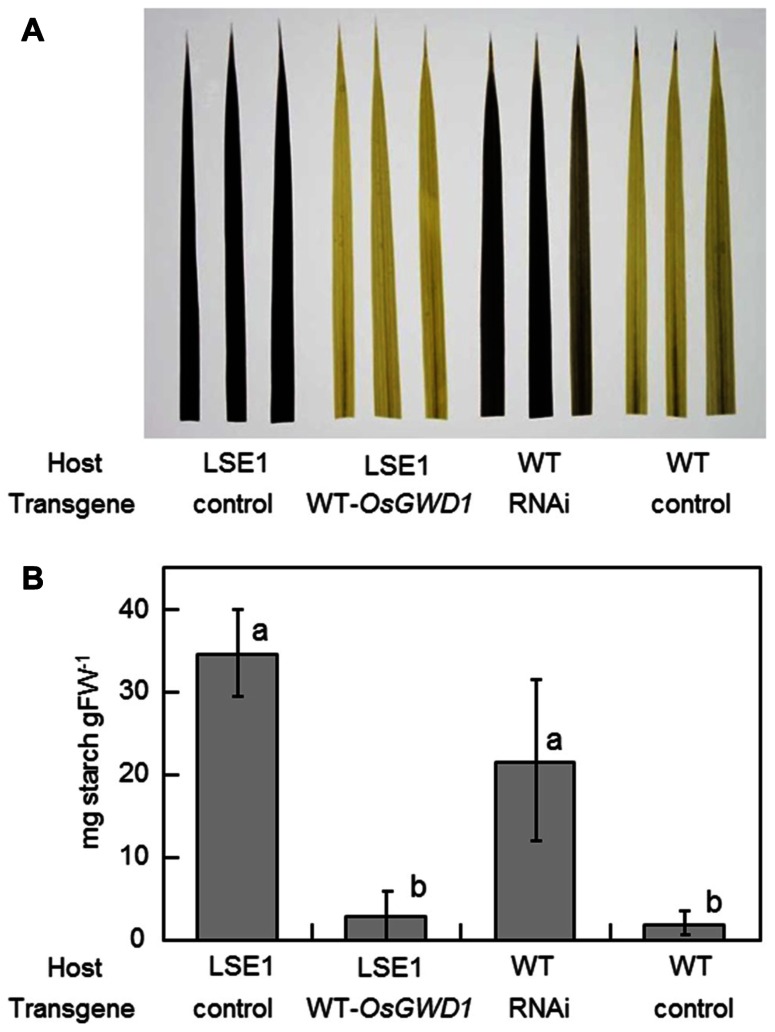
**Functional complementation of LSE1 mutation and RNAi suppression of *OsGWD1* in wild-type plants**. The uppermost fully elongated leaf blades were sampled from the transgenic rice plants at the panicle formation stage. The combinations of the host and the transgene are specified below the panel. Empty vectors were introduced as the controls for each assay. Accumulation of starch was **(A)** visualized by iodine in three independent transgenic plants or **(B)** determined enzymatically using the samples from six independent transgenic plants for each combination. Data represent the mean ± SE (*n* = 6) and different letters indicate significant differences (*p* < 0.05, Tukey’s test).

To examine whether *OsGWD1* is responsible for the LSE1 mutation, the mutant plants were introduced with a genomic DNA fragment from Nipponbare containing the entire ORF along with a 2.2-kb sequence upstream of the putative translation start point of *OsGWD1*. As expected, it was observed that *OsGWD1* from Nipponbare restored the LSE1 phenotype to wild-type; the starch content in the leaf blades of the transgenic LSE1 plants was lower and was similar to that seen in the controls (**Figure 5**). In addition, when the RNAi construct for *OsGWD1* was introduced into Nipponbare plants, the starch level in the leaf blades increased significantly compared to that in the controls, mimicking the phenotype of LSE1 (**Figure 5**).

### PHOSPHORYLATION STATUS OF STARCH IN LSE1 MUTANT

Since GWD has been reported to play a role in the degradation pathway of starch, by catalyzing the phosphorylation of the C6 position of the glucosyl residue in amylose chains (see Blennow et al., 2002 for a review), we examined the extent of phosphorylation of the glucosyl residues in starch, by measuring the amount of G6P as well as that of glucose in digested starch extracted from mature leaf blades. Leaf samples were harvested 2 h after the sunset, during which the mobilization of leaf starch would occur actively. In wild-type leaves, the percentage of phosphorylated glucosyl residues in the starch molecules was 0.20 ± 0.04% (average ± standard error, *n* = 4), namely, 2 of every 1000 glucosyl residues were phosphorylated, which is comparable with the reported phosphorylation status of starch in *Arabidopsis* and potato plants (Yu et al., 2001; also see Blennow et al., 2002 for a review). In LSE1 leaves, the percentage of phosphorylation was 0.05 ± 0.02%, significantly lower than the wild-type leaves (*p* < 0.001 by *t*-test, *n* = 4).

### VEGETATIVE GROWTH, LEAF PHOTOSYNTHESIS, AND ^13^C-PHOTOASSIMILATE PARTITIONING

**FIGURE 6:**
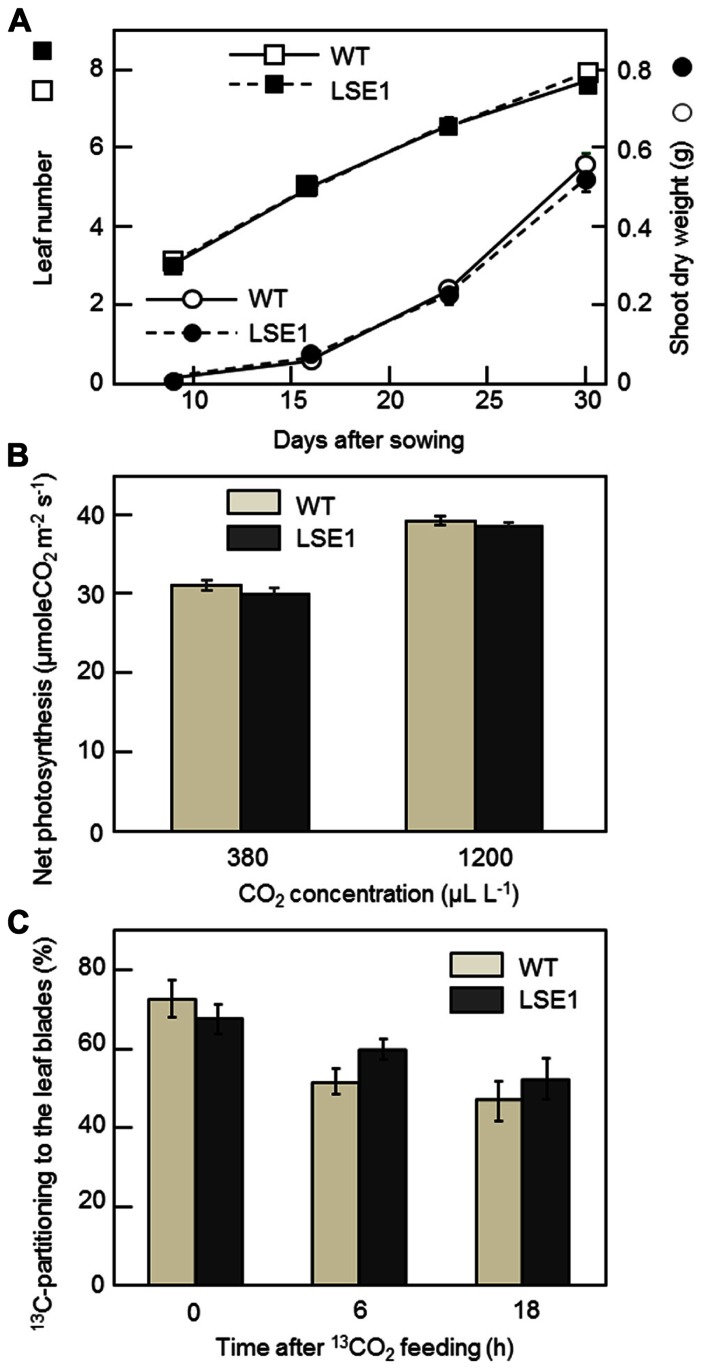
**Growth, photosynthesis and ^13^C-photoassimilate partitioning in the seedlings of LSE1**. **(A)** The dry weight of the terrestrial portion (circles) and the leaf number (squares) were examined in the seedlings of LSE1 (closed symbols) and wild-type plants (open symbols) during the early vegetative growth stage. Data represent the mean ± SE (*n* = 5). **(B)** Net photosynthesis rates of the uppermost fully elongated 7th leaf blades were measured under CO_2_ concentrations of both 380 and 1200 μL L^-^^1^. Light intensity was set at 1200 μmol photon m^-^^2^ s^-^^1^. Data represent the mean ± SE (*n* = 4). **(C)** Partitioning of ^13^C-photoassimilate to the leaf blades was measured immediately (0 h), 6 h, and 18 h after the cessation of 2-h feeding of ^13^CO_2_. The seedlings at the 6th leaf stage were used in the experiment. Data represent the mean ± SE (*n* = 4).

The vegetative growth of LSE1 was assessed by the leaf number on the main stem and the shoot dry weight. For both growth parameters, there was no significant difference between LSE1 and the wild-type control (**Figure 6A**). The rates of net photosynthesis in the leaf blades, measured under either ambient or saturating concentrations of CO_2_, were not significantly different between LSE1 and the wild-type (**Figure 6B**). The effect of the LSE1 mutation on the photoassimilate partitioning was accessed by ^13^C-tracer technique. Just after the cessation of the feeding (0-h), approximately 70% of the ^13^C remained in the leaf blades in both genotypes, and then it decreased gradually. The ^13^C-partitioning to the leaf blades at 6- and 18-h after feeding showed a tendency to be greater in LSE1 than in the wild-type, but the differences were not significant (**Figure 6C**).

### YIELD TRAITS OF LSE1

To investigate the impact of the LSE1 mutation on grain productivity in rice plants, LSE1, wild-type, and the original cultivar Nipponbare were grown in paddy fields in Tokyo and Niigata, Japan, from 2009 to 2012. Essentially the same results were obtained in all the field trials, and **Table 2** shows a representative result obtained in 2010 in Tokyo. Among the yield components of rice, the panicle number per hill, percentage of ripened grain, and 1000-grain weight were significantly lower in LSE1 plants than in wild-type and Nipponbare plants. As a result, grain yield reductions of 20~40% were observed in LSE1 compared with the control lines, in all the field trials conducted.

**Table 2:** Yield components and grain yield of LSE1, wild-type and the original cultivar Nipponbare grown under field conditions.

	Panicle number per plant	Spikelet number per panicle	Percentage of (g 1000 grains^-1^)	Grain weight	Grain yield (g m^-2^)
Wild-type	9.5 ± 0.2 (100)	105.8 ± 3.0 (100)	74.8 ± 1.6 (100)	26.5 ± 0.3 (100)	444.1 ± 8.9 (100)
LSE1	8.7 ± 0.3* (91)	101.1 ± 4.0 (96)	64.1 ± 2.0** (86)	25.1 ± 0.2** (95)	313.2 ± 21.2** (71)
Nipponbare	9.0 ± 0.3 (95)	115.8 ± 4.8 (109)	78.9 ± 0.8 (105)	26.7 ± 0.1 (101)	488.9 ± 31.0 (110)

Data were obtained in the field trial, conducted at The University of Tokyo in 2010. The numbers in parentheses indicate relative values when the mean value in the wild-type is 100. Data represent the mean values ± SE (*n* = 12). Asterisks indicate the significance of the mean values of LSE1 plants or Nipponbare plants from that of wild-type plants by *t*-test at *p < 0.05 and **p < 0.01. Note that no significant differences were observed in yield components and grain yield between wild-type plants and the original cultivar Nipponbare, indicating that these two lines were similar with regard to growth and productivity in paddy fields.

## DISCUSSION

To date, a number of mutants have been reported for the hyperaccumulation of starch in their leaves. In maize, several mutant lines exhibiting the LSE phenotype have already been reported, for example, Sxd1 (Russin et al., 1996), Tdy1 (Braun et al., 2006), and Psc (Slewinski and Braun, 2010), all of which are related to photoassimilate export from the leaves. In rice, however, no such mutation has been documented so far; to our knowledge, LSE1 is the first such case. Gene mapping and gene structure analysis revealed that the LSE1 mutant has a deletion of as much as 3.4 kb in *OsGWD1*, a putative gene for α-glucan water dikinase, and consequently, accumulates aberrant mRNA lacking one-third of the coding region in the leaf blades (**Figure 3**). Therefore, LSE1 was assumed to be a loss-of-function mutant of *OsGWD1*. This hypothesis was strongly supported by the complementation test for the LSE1 mutation with an intact *OsGWD1* and by RNAi-mediated suppression of *OsGWD1* in Nipponbare plants (**Figure 5**). Disruption of the genes for GWD has already been reported to lead to the LSE phenotype in *Arabidopsis* (SEX1; Caspar et al., 1991), *L. japonicus* (Vriet et al., 2010), and tomato (Nashilevitz et al., 2009), all of which are observed to show LSE phenotype.

In normal plants, a higher level of the *OsGWD1* transcript was observed in leaf blades and sheaths but a much lower level was seen in roots (**Figure 4A**), showing agreement with the pattern of starch accumulation in LSE plants visualized by iodine staining (**Figure 1**). The transcript level of *OsGWD1* in leaf blades changed diurnally and peaked just after the onset of the dark period (**Figure 4B**), which seems reasonable as it is believed to play a role in the degradation of starch accumulated during the light period. A similar diurnal change in the GWD transcript level has been reported in *Arabidopsis* (Yu et al., 2001; Smith et al., 2004). However, GWD protein level was shown not to change diurnally (Yu et al., 2001; Lu et al., 2005). Further investigation including diurnal change in GWD activity is needed to better understand this problem. The biochemical and physiological functions of GWD have been extensively studied in the *Arabidopsis* SEX1 mutant and are now believed to involve phosphorylation of the glucosyl residues in starch, which facilitates amylolytic degradation (see Blennow et al., 2002 for a review). In the present study, it was observed that leaf starch from the LSE1 plants contained less G6P than that from wild-type plants, consistent with observations in *Arabidopsis* SEX1 (Yu et al., 2001). Accordingly, *OsGWD1* appears to fulfill the same role in the process of leaf starch degradation in rice as in other plant species in which disruption mutants of GWD genes are reported. In other words, it was suggested that GWD has universal importance in the degradation of leaf starch among higher plants.

While LSE1 shares considerable similarity in its phenotype with the reported mutants of GWD, a conspicuous difference was found in the vegetative growth; LSE1 grows normally unlike the GWD mutants of *Arabidopsis* and *L. japonicus* (**Figure 6A**). The reason for this difference is not clear. However, it may be relevant to the physiological priority of leaf starch in the photoassimilates. In rice leaf blades, newly fixed carbon is preferentially directed to sucrose biosynthesis rather than starch, in contrast to other plant species such as *Arabidopsis*. This is evident from the fact that the content of starch and sucrose in rice leaf blades at the end of the day is ca. 5 and 20 mg per gram flesh weight (gFW^-^^1^), respectively, while that reported for *Arabidopsis* in the literatures is around 10 and 1 mg gFW^-^^1^, respectively (Figures 2A,B; e.g., Chia et al., 2004). Accordingly, rice may have sufficient capacity for the synthesis of sucrose to achieve normal growth without the transient starch in the chloroplast, and thus may experience limited impact on growth when the mobility of the transient starch is impaired by a lesion in *OsGWD1*. This view is reinforced by the observation that a knockout mutant of the ADP-glucose pyrophosphorylase (AGPase) gene, *OsAPL1*, grows normally although it accumulates less than 5% of starch in the leaf blades in comparison with wild-type plants (Rösti et al., 2007). In addition, the sucrose synthesized in photosynthetic cells was considered to be exported from the source leaves of LSE1 as efficiently as wild-type, from the facts that (1) bulk concentration of sucrose in the leaf blades did not differ between the two genotypes throughout the day (**Figure 2B**), and (2) photoassimilate export estimated by decrease in the ^13^C-partitioning to the leaf blades after the tracer feeding was also comparable between the two (**Figure 6C**). Finally, the present results indicate that hyperaccumulation of starch in the source leaves does not inhibit photosynthesis in the leaves (**Figure 6B**), which has often been assumed in the research history of the regulation of photosynthesis (see Stitt, 1991 for a review). Recently Weise et al. (2012) reported in maize that RNAi suppression of a GWD gene did not affect the vegetative growth in spite of a high level of starch accumulation in leaves. This, together with our results, suggests that vegetative growth of grass species is less dependent on GWD-mediated leaf starch remobilization than the dicot species reported.

Nevertheless, it should be also noted that the grain yield of LSE1 was significantly lower than that of wild-type plants and Nipponbare because of combined suppression of yield components (**Table 2**). This finding indicates that the impairment of GWD exerted some adverse effect on reproductive growth, although such effects are not apparent in vegetative growth. It is possible that the impairment of GWD in LSE1 slightly affected growth throughout the lifecycle in a manner that was not detectable in the short-term, and the significant reduction in the yield traits might be a manifestation of the cumulative effects. Information on the effect of GWD mutation on the reproductive growth is still limited, although Andriotis et al. (2012) recently reported that mature seeds of *Arabidopsis* SEX1 mutant have less lipid content than seeds of wild-type plants due to starvation of the carbohydrate in the fruit imported from the maternal plant. Here again, mutant plants lacking leaf starch can be a good reference because they share unavailability of leaf starch in common with LSE1. However, the results reported are inconsistent; AGPase mutants lacking leaf starch, rice *apl1* (Rösti et al., 2007) and maize *agps-m1* (Slewinski et al., 2008) showed no reduction in productivity under controlled environment whereas the latter mutant did exhibit yield reduction when grown in the field (Schlosser et al., 2012). It may be possible that the adverse effects of the mutation become apparent only in the field where plants routinely experience various environmental stresses unlike in the greenhouse. It may be also assumed that in LSE1 some disorder in starch metabolism in the stem reserve was brought about by the mutation because the stems of rice accumulate starch before heading to meet a large demand of carbohydrate for grain filling and also to buffer unfavorable environmental conditions after heading (see Slewinski, 2012 for a review). In our preliminary experiment, however, the stem starch content at the heading stage did not differ between LSE1 and the wild-type plants (data not shown), although the effects of the mutation on the mobilization of stem starch after heading are yet to be elucidated. A more extensive study with an agronomical perspective is clearly needed to uncover the mechanism of yield reduction in LSE1.

In conclusion, the LSE1 mutation in rice, i.e., disruption of *OsGWD1*, resulted in a mild phenotypic change at the whole plant level, highlighting the variable physiological priority of leaf starch among plant species. In addition to LSE1, we have isolated additional Tos17 mutant lines exhibiting the LSE phenotype, and some of these LSE mutants appear to exhibit a severe phenotype with respect to growth (Hirose, Aoki, Miyao, Hirochika, unpublished). Analysis of the LSE mutants as well as the identification of the responsible genes would increase our understandings of the molecular mechanism and/or physiological significance of photoassimilate allocation in rice.

## Conflict of Interest Statement

The authors declare that the research was conducted in the absence of any commercial or financial relationships that could be construed as a potential conflict of interest.
